# Modelling CD4 T Cell Recovery in Hepatitis C and HIV Co-infected Children Receiving Antiretroviral Therapy

**DOI:** 10.1097/INF.0000000000001478

**Published:** 2017-04-13

**Authors:** Adedeji O. Majekodunmi, Claire Thorne, Ruslan Malyuta, Alla Volokha, Robin E. Callard, Nigel J. Klein, Joanna Lewis

**Affiliations:** From the * Institute of Child Health, and † CoMPLEX: Centre for Mathematics and Physics in the Life Sciences and Experimental Biology, University College London, London, United Kingdom; ‡ Perinatal Prevention of AIDS Initiative, Odessa, Ukraine; § Shupyk National Medical Academy of Postgraduate Education, Kiev, Ukraine; ¶ Great Ormond Street Hospital, and ‖ Department of Infectious Disease Epidemiology, Imperial College London, St Mary’s Campus, London, United Kingdom.

**Keywords:** pediatric hepatitis C, HIV and hepatitis C coinfection, CD4+ T cell reconstitution, nonlinear mixed-effects modeling, antiretroviral therapy

## Abstract

**Background:**

The effect of hepatitis C virus (HCV) coinfection on CD4^+^ T cell recovery in treated HIV-infected children is poorly understood.

**Objective:**

To compare CD4^+^ T cell recovery in HIV/HCV coinfected children with recovery in HIV monoinfected children.

**Method:**

We studied 355 HIV monoinfected and 46 HIV/HCV coinfected children receiving antiretroviral therapy (ART) during a median follow-up period of 4.2 years (interquartile range: 2.7–5.3 years). Our dataset came from the Ukraine pediatric HIV Cohort and the HIV/HCV coinfection study within the European Pregnancy and Paediatric HIV Cohort Collaboration. We fitted an asymptotic nonlinear mixed-effects model of CD4^+^ T cell reconstitution to age-standardized CD4 counts in all 401 children and investigated factors predicting the speed and extent of recovery.

**Results:**

We found no significant impact of HCV coinfection on either pre-ART or long-term age-adjusted CD4 counts (z scores). However, the rate of increase in CD4 z score was slower in HIV/HCV coinfected children when compared with their monoinfected counterparts (*P* < 0.001). Both monoinfected and coinfected children starting ART at younger ages had higher pre-ART (*P* < 0.001) and long-term (*P* < 0.001) CD4 z scores than those who started when they were older.

**Conclusions:**

HIV/HCV coinfected children receiving ART had slower CD4^+^ T cell recovery than HIV monoinfected children. HIV/HCV coinfection had no impact on pre-ART or long-term CD4 z scores. Early treatment of HIV/HCV coinfected children with ART should be encouraged.

## Abstract 2

Supplemental Digital Content is available in the text.

Globally, over 170 million individuals are estimated to be living with chronic hepatitis C virus (HCV) infection.^1^ Of these, an estimated 11 million are children under the age of 15.^2^ The prevalence of pediatric HCV infection varies geographically, from 0.05% in the Western world to 5.8% in resource-limited settings.^3^ As a result of shared routes of transmission, about a third of the 37 million HIV-infected individuals worldwide are thought to be coinfected with HCV.^4,5^

Most children with HCV are vertically infected, and an estimated 60,000 HCV-infected infants are born worldwide every year.^6^ A recent meta-analysis estimated the risk of mother-to-child transmission to be 6% for HCV-seropositive, RNA-positive women, increasing to nearly 11% for those with HIV coinfection.^7^ Although highly debated and only demonstrated in a few studies, additional factors contributing to increased risk of transmission may include high hepatitis C viral load and the presence of HCV in maternal peripheral blood mononuclear cells.^4,7,8^ HIV/HCV coinfected mothers receiving combination antiretroviral therapy (ART) are less likely to transmit HCV to their unborn child.^9^ The incidence of spontaneous HCV clearance varies in childhood depending on virus genotype and figures from 7.5% to 25% have been quoted in the literature.^10–12^ However, in the presence of HIV coinfection, the likelihood of spontaneous clearance is much reduced in children^13^ as in adults.^14^

The effects of HCV coinfection on CD4^+^ T cell recovery in HIV-infected patients receiving ART remain a subject of much controversy. The large number of investigations conducted in adults have yielded conflicting findings, with some studies reporting a negative effect on CD4^+^ T cell recovery^15–18^ and others reporting no effect.^19–22^

Improvements in CD4 count in response to ART are the result of viral suppression, which allows the body’s homeostatic mechanisms to repopulate the CD4^+^ T cell pool. These mechanisms vary with age, and in children are thought to rely largely on a highly active thymus, which produces large number of naive CD4^+^ T cells before undergoing involution in early adulthood.^23,24^ Thymic activity in HIV-infected children has also been observed to improve on ART.^25–27^

In this study, we aimed to improve understanding of the impact of HCV coinfection on CD4^+^ T cell recovery in children receiving ART. By using a larger study population than has previously been available, and analysis methods specifically suited to longitudinal CD4 measurements in children,^28,29^ we are able to present a fuller picture of CD4 reconstitution in HIV/HCV coinfected children.

## METHODS

### Data Sources and Eligibility

Data on coinfected children came from a study of HIV/HCV coinfection within the European Pregnancy and Paediatric HIV Cohort Collaboration (EPPICC), in 8 countries across Europe, including Ukraine. In the EPPICC HIV/HCV coinfection study, children older than 18 months of age, adolescents and young adults younger than 25 years of age were eligible for inclusion if they were infected with HIV and with chronic HCV acquired vertically or in childhood.

Data on monoinfected children came from the Ukraine Paediatric HIV Cohort Study. The Ukraine Paediatric HIV Cohort Study was established in January 2011 and enrolled HIV-infected infants, children and adolescents being cared for in 6 HIV/AIDS centers in Ukraine. This study collected anonymized demographic, clinical and laboratory data on children according to a standard protocol, with informed consent and is a member of EPPICC. The included studies were observational, with laboratory testing being conducted locally. Children with known spontaneous viral clearance of HCV (ie, disappearance of HCV RNA in ≥2 consecutive serum samples taken 6 months apart) were excluded.

### Definitions

All children were older than 18 months of age. HCV-infected children were identified by detection of positive HCV antibody and/or greater than or equal to 2 positive HCV RNA detected on 2 separate clinic visits at least 3 months apart. HIV infection in children was defined as detection of HIV antibody and/or positive HIV RNA or DNA polymerase chain reaction in a minimum of 2 samples obtained on separate visits. “Coinfected children” in this study refers to HIV/HCV coinfected children, while “monoinfected children” refers to HIV monoinfected children. Our threshold for detection of HIV viral load was set at 50 copies/mL.

### Ethical Approval

Each participating cohort in EPPICC followed local ethical guidelines. The Ukraine Paediatric HIV Cohort Study has approval from the UCL Research Ethics Committee and local institutional review boards.

### Age-adjustment of CD4 Counts

Healthy children demonstrate a pronounced fall in their CD4^+^ T cell count as they approach adulthood.^30^ Because of these age-associated changes, a direct comparison of raw CD4 counts between children of different age groups is not possible. In view of this, raw CD4 counts of our cohort were converted to age-standardized z scores. These z scores were originally calculated based on the normalized expected distribution for age-matched HIV-negative children born to HIV-positive mothers.^31^ A z score of 0 implies that a child has a normal CD4 count for their age, while scores of ±2 indicate that a child is on the 97.7% and 2.3% centiles, respectively, of the expected CD4 count for their age. Although CD4 percentage is relatively stable with age and has been widely used in published studies, we have chosen to use age-corrected CD4 counts in our analysis because CD4 percentage is influenced by CD8 T cells which are likely to be affected by HIV/HCV coinfection. Furthermore, CD4 counts have been shown to be of no less prognostic value than CD4 percentage.^32^ Children with fewer than 2 CD4 measurements were excluded from the study.

### Mixed-effects Modeling

CD4 z scores were analyzed using nonlinear mixed-effects models. Within the mixed-effects framework, we assumed that recovery of the CD4 z score followed an asymptotic pattern as described elsewhere^33^ and illustrated in Figure 1. Briefly, in this model, the z score starts at a below-healthy initial value, *int*_*i*_. Following ART initiation, the z score increases, trending in the long term to a higher, stable level, *asy*_*i*_ as described by the following equation:

**FIGURE 1.**
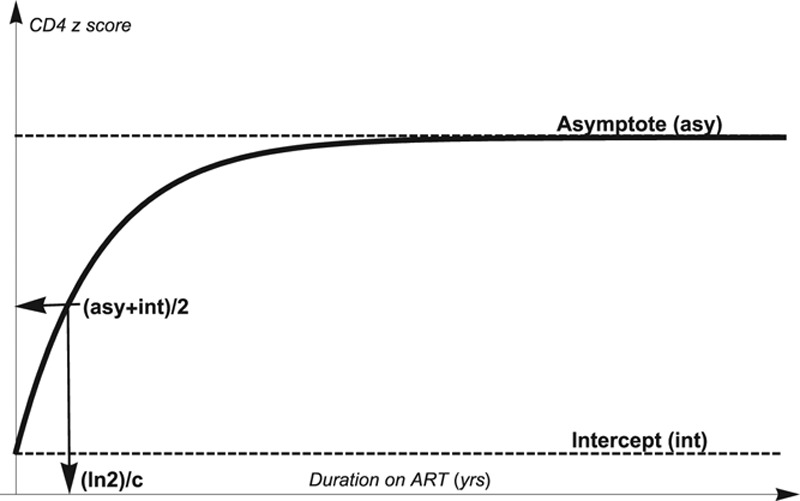
A schematic showing the mathematical model of immune reconstitution used in this study. C is the rate of recovery of age-adjusted CD4 counts. After ART, patients are expected to reconstitute their CD4^+^ T cells from an initial age-adjusted count (int) to a steady value (asy).


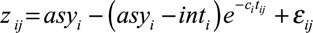


where z_*ij*_ represents CD4 z score for child *i* at time *t*_*ij*_ after starting ART; *int*_*i*_ represents CD4 z score at ART initiation for child *i*; *asy*_*i*_ represents the long-term CD4 z score for child *i*; *c*_*i*_ is a parameter that describes the rate of increase in CD4 z score for child *i*; *ln2/c*_*i*_ being the time taken to achieve half of the total recovery from *int*_*i*_ to *asy*_*i*_. The term *ε*_*ij*_ is the “residual error,” which represents measurement error, random variation and model misspecification leading to differences between the recorded data and the form of the curve in Figure 1. In previous studies, this approach has provided a good description of CD4^+^ T cell dynamics in children starting ART,^33,34^ with all 3 of the model parameters conveying a clear clinical meaning (Appendix, Supplemental Digital Content 1, http://links.lww.com/INF/C639).

### Covariate Analysis

We used forward and backward stepwise selection with exit *P* values of 0.05 and 0.01, respectively (likelihood ratio test), to investigate potential effects on CD4 recovery of: age and AIDS status at start of ART, gender, HCV status, pre-ART HIV viral load and EPPICC cohort.

### Software and Algorithms

All mixed-effects model fitting was by maximum likelihood implemented in NONMEM.^35,36^ Further data analysis was done in R (R Foundation for Statistical Computing, Vienna, Austria)^37^ and predictions generated from the model were plotted in Wolfram Mathematica.^38^

## RESULTS

Characteristics of the study population are described in Table 1. A total of 355 HIV monoinfected and 46 coinfected children were included for analysis. Median age at start of ART was 3.1 years [interquartile range (IQR): 1.31–5.65 years] for the coinfected group and 4.4 years (IQR: 1.73–7.07 years) for the monoinfected group. Median year of birth was 2004 (IQR: 2001–2006, range: 1989–2011).

**TABLE 1.**
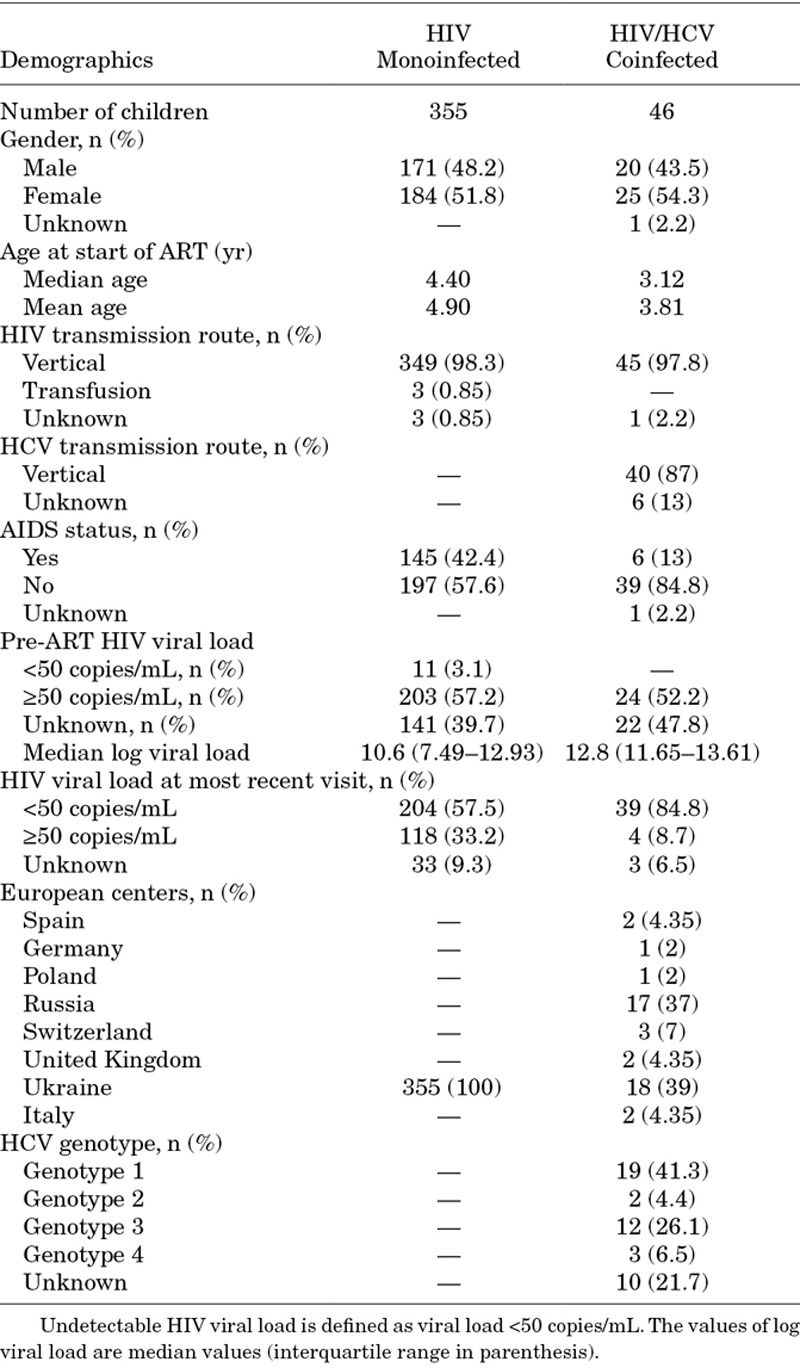
Characteristics of Study Population

The monoinfected children were all from Ukraine, while the coinfected children were from 8 countries across Europe including Ukraine. The median number of CD4 measurements available per child was 5 for the entire population (IQR: 3–7) as well as for the monoinfected children (IQR: 4–7) and 4 for the coinfected children (IQR: 3–6). Median follow-up period on ART was 4.2 years for the entire population (IQR: 2.7–5.3 years), 4.1 years for the monoinfected children (IQR: 2.7–5.2 years) and 5.1 years for the coinfected children (IQR: 3.1–5.6 years). Figure 2 shows the overall trend in average CD4 z scores for all 401 children plotted against time on ART.

**FIGURE 2.**
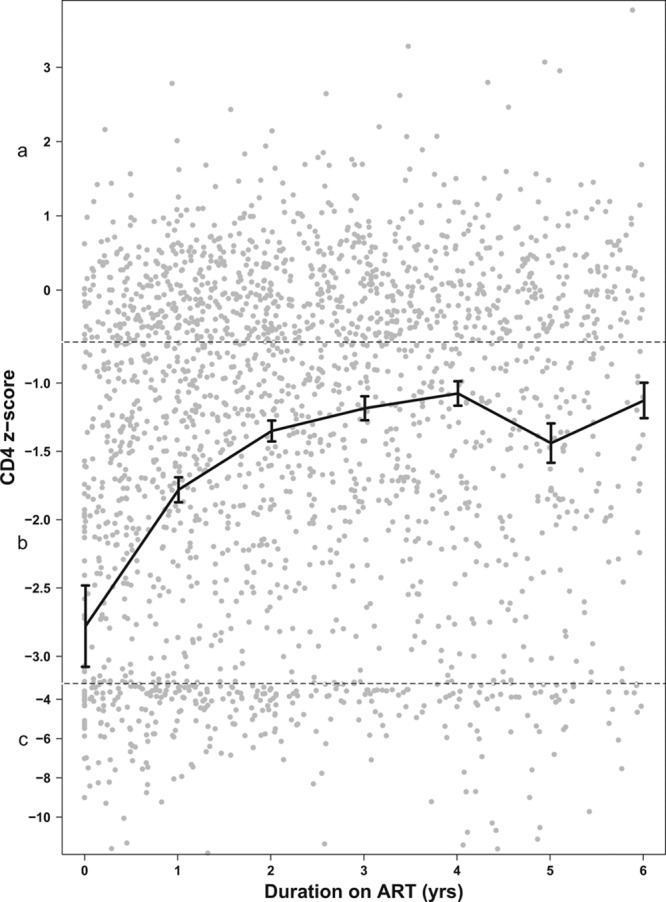
Observed age-adjusted CD4 counts (z scores) plotted against time on ART for all 401 children included in this study. This plot has been scaled into 3 different windows using dotted lines to highlight the extent of immune recovery within the data. A: Observed age-adjusted CD4 counts between −0.7 and 3.9 plotted against duration on ART. B: Observed age-adjusted CD4 counts (z scores) between −3.2 and −0.7 plotted against time on ART. The black line through the data represents the mean CD4 z scores at yearly intervals while the bars represent standard errors on the mean CD4 z scores. C: Observed age-adjusted CD4 counts between −11.9 and −3.2 plotted against duration on ART.

### HIV Viral Load

The data provided here were based on the latest HIV viral load reported in each child. These data were available for 43 of the 46 (94%) coinfected children, 39 (91%) of whom had undetectable HIV viremia at their latest blood test. Of the 355 HIV monoinfected children, data on latest HIV viral load were available on 322 (91%) children, 204 (63%) of whom had undetectable viral load. The 118 monoinfected children with detectable HIV viral load had a median log viral load of 5.98 log copies/mL (IQR: 4.6–9.0). Median duration on ART at the time of latest HIV viral load was 3.1 years for all 365 children combined (IQR: 1.5–4.6 years), 2.9 years for the monoinfected children (IQR: 1.5–4.3 years) and 5.4 years for the coinfected children (IQR: 2.6–7.5 years).

### HCV Viral Load

The data on HCV viral load were only available in 23 coinfected children. Two different thresholds were identified among patients with undetectable HCV viremia: 3200 and 200 copies/mL, probably reflecting the use of different assays.

### Anti-HCV and ART

All 10 HIV/HCV coinfected children who received anti-HCV therapy had pegylated-interferon and ribavirin. Of these, 6 failed treatment, while 3 achieved spontaneous viral response. Treatment outcome was unknown for 1 child. Data on ART regimen were available in 35 of 46 HIV/HCV coinfected children and in all 355 monoinfected children. By far the most common ART drugs were lamivudine, zidovudine and kaletra (lopinavir and ritonavir). A total of 33% of monoinfected children were on a 3-ART regimen compared with 49% in the coinfected group.

### Factors Determining Pre-ART and Long-term CD4 Z Score

We found that on average (fixed effects), children started ART with a CD4 z score of −2.42, corresponding to the 0.78th centile in uninfected children of the same age. Pre-ART z score was 0.32 units lower per year older at ART initiation, and there was also an effect of cohort, with children from the United Kingdom and Italy starting ART with lower CD4 counts for their age and the children in the Spanish cohort starting with higher CD4 z scores (complete effect sizes are provided in Table 2). The only predictor of long-term CD4 level for age was the age at ART initiation: long-term z score was 0.11 units lower per year older at ART initiation. The average long-term z score from the final model was −1.07, which corresponds to the 14th centile.

**TABLE 2.**
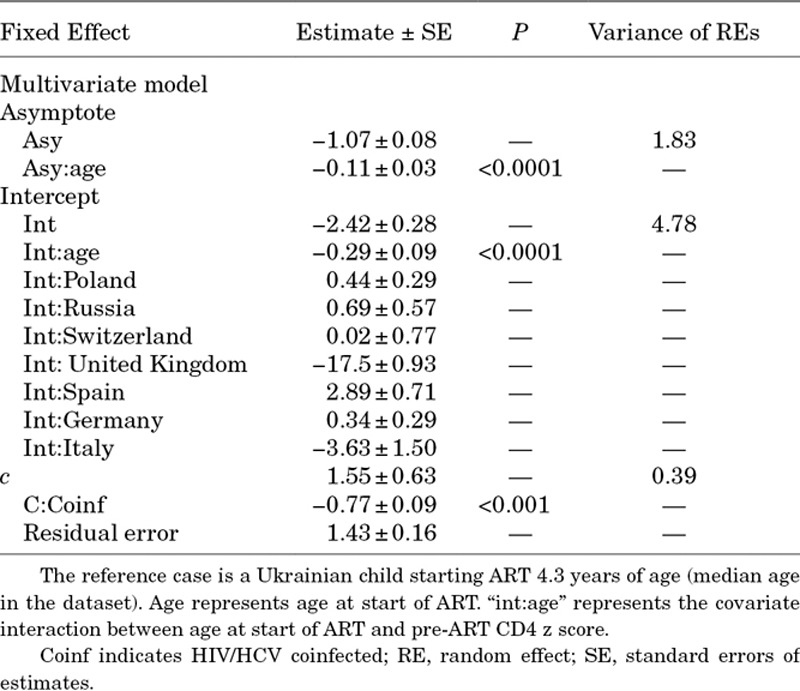
Parameter Estimates for the Final Multivariate Model

Figure 3A shows model predictions (fixed effects) for 3 categories of HIV monoinfected children at 2, 4 and 8 years. As expected, younger monoinfected children commence ART at higher age-adjusted CD4 counts and also achieve higher long-term values of CD4 z scores when compared with older children.

**FIGURE 3.**
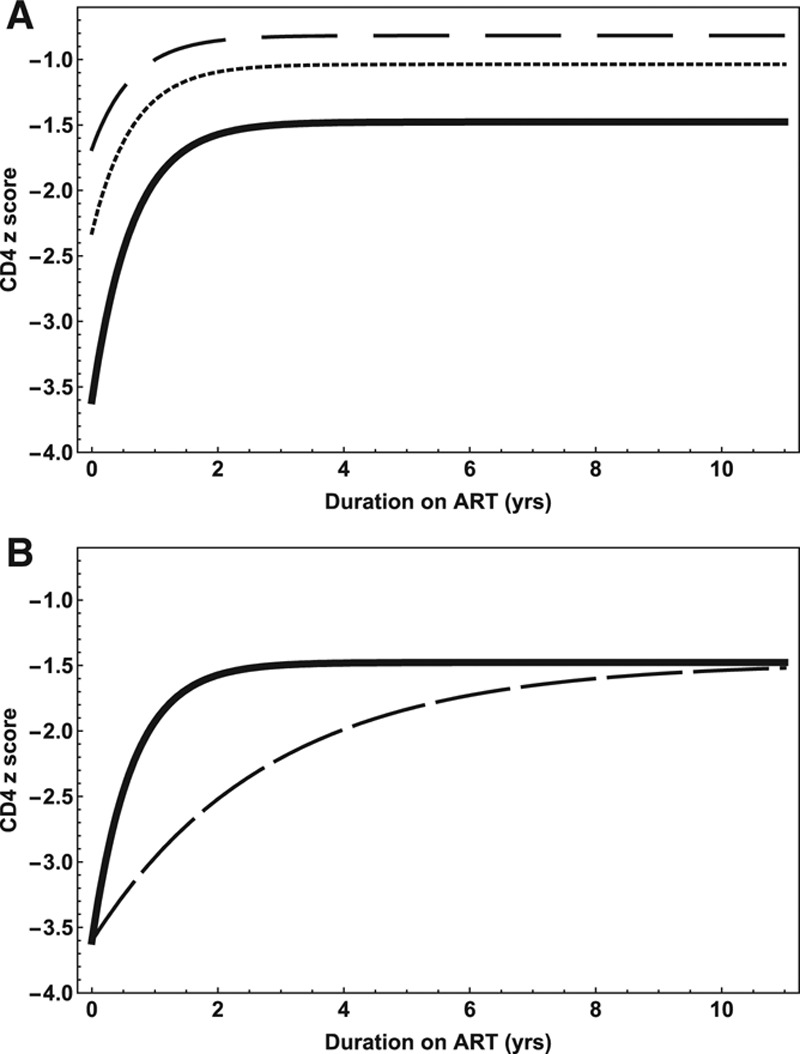
Model predictions for CD4^+^ T cell reconstitution in coinfected and monoinfected children. A: Model recovery profiles (fixed effects) predicted for a 2-year-old (long dashed lines), 4-year-old (short dashed lines) and an 8-year-old (solid line) child (all HIV monoinfected). Younger monoinfected children start ART at higher intercept values and also achieve higher long-term values of age-adjusted CD4 counts when compared with older children. B: Fixed effect profiles predicted for a monoinfected (solid line) and a coinfected (long dashed line) 8-year-old child. The HIV/HCV coinfected child is seen to have a significantly slower rate of increase in age-adjusted CD4 relative to the monoinfected child. However, both predictions have comparable intercept and asymptote values of their age-adjusted CD4 counts.

### Hepatitis C Coinfected Children Have a Slower Rate of Increase in CD4 Z Score Than HIV Monoinfected Children

HCV coinfection was a significant predictor of the rate of CD4 z score recovery (denoted *c* in Equation 1). We found that coinfected children had a significantly reduced recovery rate of 0.357 per year compared to 1.55 per year in monoinfected children (Fig. 2B). This difference corresponds to a time for half the long-term recovery to occur of 2 years in coinfected children, compared with 5 months (0.45 years) in monoinfected children. Interestingly, we found no statistically significant effect of HCV coinfection on either pre-ART or long-term CD4 z scores (*P* = 0.80 or *P* = 0.77, respectively), suggesting that although recovery was slower in coinfected children, they started ART with similar CD4 counts, and on long-term therapy do eventually achieve similar CD4 levels to their monoinfected peers.

### Pre-ART HIV Viral Load Had No Impact on Immune Recovery

To investigate the effect of pre-ART viral load on CD4 recovery, we analyzed a subset of our data consisting of 238 children who had pre-ART HIV viral load data. We compared the covariate model selection process with pre-ART HIV viral load excluded or included in addition to the other 5 covariates (age, AIDS status at start of therapy, gender, HCV status and EPPICC cohort). Both model selection processes retained only covariate relationships between asymptote, intercept and age at start of ART. There was thus no evidence that pre-ART HIV viral load predicts either long-term CD4 z scores or rate of recovery (*c*) once other factors have been taken into account, although this may be a limitation of the data available.

## DISCUSSION

In this study, we investigated the impact of HCV coinfection on CD4^+^ T cell reconstitution in HIV-infected children receiving ART. By fitting a nonlinear mixed-effects model to longitudinal data from 401 children, we found that HIV/HCV coinfected children had significantly slower recovery of their age-adjusted CD4 counts than HIV monoinfected children. Despite this reduced rate of recovery, the coinfected children still managed to achieve long-term CD4^+^ T cell levels comparable to HIV monoinfected children. Our fixed effect estimates for rate of recovery (*c*), pre-ART and long-term CD4 z scores are consistent with others obtained in a different multicenter European pediatric study.^33^

In a recent paper by Marcus et al,^39^ it was shown that HIV/HCV coinfected adults have delayed CD4^+^ T cell reconstitution on ART, relative to their monoinfected counterparts. Similarly, a meta-analysis conducted by Tsiara et al^40^ in 2013 of 21 studies involving 22,533 adult patients reported that even though HCV had a demonstrable adverse effect on immune reconstitution in the first 2 years of ART, this trend was not sustained in the long term. Our findings in children (Fig. 3B) add to this by suggesting that this transient adverse effect of HCV coinfection in adults might be explained by a reduced rate of increase in CD4^+^ T cell recovery.

To the best of our knowledge, this is the first large-scale mathematical modeling analysis of the effect of HCV on CD4^+^ T cell recovery in HIV/HCV coinfected children. Our findings are consistent with those of Micheloud et al,^41^ whose smaller 2007 study found similar long-term CD4^+^ T cell recovery in 19 coinfected and 25 monoinfected children.

A number of mechanisms could be driving the slower rate of reconstitution in HCV coinfected children receiving ART. One possible explanation is reduced thymic output. Some studies have already shown that HCV monoinfection and HIV/HCV coinfection appear to negatively affect thymic output in adult patients.^19,42^ In children however, there is evidence that thymic output may recover on ART and this might explain why long-term CD4 counts were unaffected by HCV coinfection in this study.^27,43^ Another possible mechanism that could give rise to slower CD4^+^ T cell recovery in HIV/HCV coinfected individuals is increased T cell activation.^44^ It has been suggested that increased T cell activation may lead to immune exhaustion through excessive cytokine production, which then impairs CD4^+^ T cell recovery,^44^ and effective treatment of HCV infection with interferon and ribavirin has been seen to reduce T cell activation.^45^ A third hypothesis is that a poorer virologic response in coinfected children means that CD4 count recovers more slowly. This is difficult to investigate in our cohorts, for whom viral load data are sometimes patchy, but could be investigated in other children in future studies.

One of the major strengths of this work is the number of HIV/HCV coinfected children included for analysis, which is more than double the number included in the only previous analysis.^41^ Furthermore, our mixed-effects approach provides a statistically rigorous method appropriate for longitudinal datasets where observations from an individual are often correlated.^46^

A limitation of the study lies in the fact that all the HIV monoinfected children were from Ukraine, whereas the HIV/HCV coinfected children were selected from 8 countries across Europe (including Ukraine). Hence, laboratory testing was conducted locally. To take account of this, we have added the effect of cohort as a covariate in informing model predictions. An additional limitation was that the effect of pre-ART HIV viral load could be investigated only in the subset of the children in whom it was available (238 children, 59%). Nonetheless, there was no evidence of an effect of pre-ART HIV viral load on CD4^+^ T cell recovery. Furthermore, not all the latest HIV viral loads were measured after the same duration of ART. This is likely to account for the greater proportion of coinfected children with viral suppression. Finally, we were unable to explore the impact of HCV treatment on CD4^+^ T cell reconstitution because of the small number of children treated.

The delayed CD4^+^ T cell recovery seen in HIV/HCV coinfected children suggests that early treatment with ART may be more critical in coinfected children. If our aim is to minimize the time for which children are exposed to the increased risk of opportunistic infection associated with very low CD4 counts, it may be necessary to start therapy earlier in coinfected children because they will take longer to return to “safer” CD4 levels. As illustrated in Figure 2B, coinfected children take more than 4 times as long as monoinfected children to recover their CD4 levels. This adds to the case for early ART in coinfected children that has already been made: that good control of HIV viremia is likely to lead to a better clearance of HCV,^47^ and reinforces the latest paediatric European network for treatment of AIDS (PENTA) guideline for treatment of pediatric HIV, which has diagnosis of HCV coinfection as a definite indication for commencing ART in children.^48^

The findings in this study support earlier work^33^ demonstrating the importance of age on CD4^+^ T cell reconstitution. While our study did not find evidence of any long-term effect of HIV/HCV co-infection on CD4^+^ T cell recovery, the slower rate of recovery does suggest that HIV/HCV coinfection may have other, as yet unidentified, immunologic consequences. Further studies of vertically HIV/HCV coinfected children are, therefore, needed to fully understand the implications of HCV coinfection on immune recovery. Additional studies on the efficacy of the new direct-acting antivirals are needed to fully understand whether our findings will persist after their introduction in children.

## ACKNOWLEDGMENTS

### We are very grateful to the children and the families who contributed to the study.

The HIV/HCV Co-infection Study Group in EPPICC—participating cohorts: Belgium: Hospital St Pierre Cohort, Brussels: T. Goetghebuer, M. Hainaut, E. Van Der Kelen; Germany: German Competence Network on HIV infected children: C. Koenigs, K. Mantzsch; Italy: Italian Register for HIV-infection in Children: M. de Martino, L. Galli, E. Venturini (Florence), participating sites: Dr. V. Giacomet (Milan), Dr. L. Nicolini, Dr. Del Puente (Genoa), Dr. C. Gabiano (Turin), Dr. A. Guarino (Naples), Dr. S. Martinazzi (Brescia), Dr. A. Miniaci (Bologna); Poland: Medical University Warsaw/Regional Hospital of Infectious Disease cohort: Dr. S. Dobsz, Prof. M. Marczynska; Romania: “Victor Babes” Hospital Cohort, Romania: Dr. L. Ene, Dr. D. Duiculescu; Russia: Republican Hospital of Infectious Diseases (RHID), St Petersburg cohort: Dr. M. Miloenko, Dr. K. Dodonov, Dr. I. Latysheva, Prof. E. Voronin; Spain: Cohort of the Spanish Paediatric HIV Network (CoRISpe) (Madrid and Barcelona): Dr. P. Rojo, Dr. A. Noguera-Julian; Switzerland: Swiss Mother and Child HIV Cohort Study (MoCHiV): Dr. C. Rudin, Dr. A. Duppenthaler; United Kingdom: UK Collaborative HIV Paediatric Study (CHIPS): Dr. A. Turkova, Dr. A. Judd; Ukraine: Ukraine Cohort of HIV-infected children: Dr. R. Malyuta, Dr. A. Volokha, Dr. C. Thorne. The Ukraine Paediatric HIV Cohort Study in EuroCoord: Dr. T. Kaleeva, Dr. Y. Baryshnikova (Odessa Regional Centre for HIV/AIDS), Dr. S. Soloha (Donetsk Regional Centre for HIV/AIDS), Dr. N. Bashkatova (Mariupol AIDS Center), Dr. I. Raus (Kiev City Centre for HIV/AIDS), Dr. O. Glutshenko, Dr. Z. Ruban (Mykolaiv Regional Centre for HIV/AIDS), Dr. N. Prymak (Kryvyi Rih) and Dr. G. Kiseleva (Simferopol).

EuroCoord Executive Board: Fiona Burns, University College London, United Kingdom; Geneviève Chêne, University of Bordeaux, France; Dominique Costagliola (Scientific Coordinator), Institut National de la Santé et de la Recherche Médicale, France; Carlo Giaquinto, Fondazione PENTA, Italy; Jesper Grarup, Region Hovedstaden, Denmark; Ole Kirk, Region Hovedstaden, Denmark; Laurence Meyer, Institut National de la Santé et de la Recherche Médicale, France; Heather Bailey, University College London, United Kingdom; Alain Volny Anne, European AIDS Treatment Group, France; Alex Panteleev, St. Petersburg City AIDS Centre, Russian Federation; Andrew Phillips, University College London, United Kingdom; Kholoud Porter, University College London, United Kingdom; Claire Thorne, University College London, United Kingdom.

EuroCoord Council of Partners: Jean-Pierre Aboulker, Institut National de la Santé et de la Recherche Médicale, France; Jan Albert, Karolinska Institute, Sweden; Silvia Asandi, Romanian Angel Appeal Foundation, Romania; Geneviève Chêne, University of Bordeaux, France; Dominique Costagliola (chair), INSERM, France; Antonella d’Arminio Monforte, ICoNA Foundation, Italy; Stéphane De Wit, St. Pierre University Hospital, Belgium; Peter Reiss, Stichting HIV Monitoring, Netherlands; Julia Del Amo, Instituto de Salud Carlos III, Spain; José Gatell, Fundació Privada Clínic per a la Recerca Bíomèdica, Spain; Carlo Giaquinto, Fondazione PENTA, Italy; Osamah Hamouda, Robert Koch Institut, Germany; Igor Karpov, University of Minsk, Belarus; Bruno Ledergerber, University of Zurich, Switzerland; Jens Lundgren, Region Hovedstaden, Denmark; Ruslan Malyuta, Perinatal Prevention of AIDS Initiative, Ukraine; Claus Møller, Cadpeople A/S, Denmark; Kholoud Porter, University College London, United Kingdom; Maria Prins, Academic Medical Centre, Netherlands; Aza Rakhmanova, St. Petersburg City AIDS Centre, Russian Federation; Jürgen Rockstroh, University of Bonn, Germany; Magda Rosinska, National Institute of Public Health, National Institute of Hygiene, Poland; Manjinder Sandhu, Genome Research Limited; Claire Thorne, University College London, United Kingdom; Giota Touloumi, National and Kapodistrian University of Athens, Greece; Alain Volny Anne, European AIDS Treatment Group, France.

EuroCoord External Advisory Board: David Cooper, University of New South Wales, Australia; Nikos Dedes, Positive Voice, Greece; Kevin Fenton, Public Health England, US; David Pizzuti, Gilead Sciences, US; Marco Vitoria, World Health Organization, Switzerland.

EuroCoord Secretariat: Silvia Faggion, Fondazione PENTA, Italy; Lorraine Fradette, University College London, United Kingdom; Richard Frost, University College London, United Kingdom; Andrea Cartier, University College London, United Kingdom; Dorthe Raben, Region Hovedstaden, Denmark; Christine Schwimmer, University of Bordeaux, France; Martin Scott, UCL European Research & Innovation Office, United Kingdom.

## Supplementary Material
