# Supplementary material for: Modelling CD4 T Cell Recovery in Hepatitis C and HIV Co-infected Children Receiving Antiretroviral Therapy
Source: Pediatr Infect Dis J. 2017 Apr 13;36(5):e123–9. doi: 10.1097/INF.0000000000001478 (PMC5380220; doi:10.1097/INF.0000000000001478)
Supplement: Supplementary file 1 [file inf-36-e123-s001.docx]

# Appendix

***Mixed effect models***

Mixed effects models are a statistical framework for longitudinal data analysis that provide a picture of the CD4^+^ T cell response to treatment both in the population as a whole, on average, and in each child individually. This enables quantification of the differences between children attributable to differences in physiology or environment. Mixed effects modelling makes it possible to fit flexible models to longitudinal data with missing values. In addition, the effects of covariates of interest can be investigated. In our mixed-effects model, an individual child’s response to time on ART is assumed to follow a trajectory as described by equation (1) in the main text. For each child, the parameters of the model are a combination of a population-average value (the “fixed effect”) and a child-specific deviation from this average (the “random effect”):

${int}_{i}={int}_{i}^{f}+{int}_{i}^{r}$	${asy}_{i}={asy}_{i}^{f}+{asy}_{i}^{r}$	$c_{i}=c_{i}^{f}e^{c_{i}^{r}}$

where superscripts *f* and *r* represent fixed and random effects, respectively. The fixed effects are modeled as a linear combination of covariate effects:

$${\left( {int}_{i}^{f}, {asy}_{i}^{f},c_{i}^{f} \right)\boldsymbol{=A}}_{\boldsymbol{i}}\boldsymbol{\beta}$$

where $\boldsymbol{\beta}$ is a vector of parameters to be inferred. $\boldsymbol{A}_{\boldsymbol{i}}$ is a design matrix which links the observations to the fixed effects, as described in the section below (see section titled “covariate model”).

The vector of random effects, $\boldsymbol{u}_{\boldsymbol{i}}\boldsymbol{=}\left( {int}_{i}^{r}, {asy}_{i}^{r},c_{i}^{r} \right)$has a multivariate normal distribution with a mean of 0 and independent of the residual errors ($\epsilon_{ij}$):

$\boldsymbol{u}_{\boldsymbol{i}}\boldsymbol{\sim}N\boldsymbol{(}0\boldsymbol{,}\boldsymbol{\Sigma}\boldsymbol{)}$

The residual errors denoted in Equation (1) by $\epsilon_{ij}$ are also assumed to be normally distributed with a mean of 0 and variance $\sigma^{2}$:

$$\epsilon_{ij} \sim N\left( 0, \sigma^{2} \right)$$

***Covariate Model***

The final model obtained from the stepwise covariate selection (see Table 2 for more details) can be expressed mathematically as:

$$\left( \begin{matrix} {asy}_{i}^{f} \\ {int}_{i}^{f} \\ c_{i}^{f} \end{matrix} \right)= \left( \begin{matrix} {asy}_{0i} & {asy}_{A_{gi}} & 0 \\ {int}_{0i} & {int}_{A_{gi}} & 0 \\ c_{0i} & 0 & c_{Hi} \end{matrix} \begin{matrix} 0 \\ {int}_{Ei} \\ 0 \end{matrix} \right)\left( \begin{matrix} 1 \\ \begin{matrix} A_{gi} \\ \begin{matrix} H_{i} \\ E_{i} \end{matrix} \end{matrix} \end{matrix} \right)$$

where $A_{gi}$, $H_{i}$ and $E_{i}$, each represent age at start of ART, HCV status, EPPICC center respectively. The asymptotic model in equation (1) was fitted with random effects added to all three parameters (${asy}_{i}$, ${int}_{i}$ and $c_{i}$).

***Covariance Matrix***

The covariance matrix of the mixed-effects model, which describes the variability within the population in pre-ART and long-term z-scores and their correlation is given below:

In a separate analysis, the final covariate model described above was modified to include correlation between pre-ART and long-term CD4 z-scores. We found that the inclusion of this correlation made no significant difference to the overall fit of the model.

***Age-adjusted CD4 counts***

One drawback of the CD4 z-score function is that it does not allow the youngest children to have very low z-scores making it very unstable at low CD4 counts. Hence, low z-scores do not give reliable information for age and based on previous sensitivity analyses, a total of 28 CD4 z-scores below -12 were excluded from our analysis^33^.

**Supplementary Figure 1**

Top: boxplots illustrating the trend in age at start of ART (top left) between mono-infected and co-infected patients. Mono-infected children generally started ART much later than co-infected children. HIV/HCV coinf: HIV/HCV co-infected children, HIV Monoinf: HIV mono-infected children.

Bottom left: diagnostic plot showing good agreement between predicted CD4 z scores and observed CD4 z scores. Bottom right: diagnostic plot inspecting trends in residual error with predicted CD4 z-score. As expected for a good predictive model, no trend is apparent.
